# Visualization of non-Newtonian convective fluid flow with internal heat transfer across a rotating stretchable surface impact of chemical reaction

**DOI:** 10.1038/s41598-022-14384-7

**Published:** 2022-06-21

**Authors:** Zeeshan, Ilyas Khan, Nosheen Feroz, Fuad S. Al-Duais, Omar Mahmoud

**Affiliations:** 1 Department of Mathematics and Statistics, Bacha University Charsadda, Charsadda, KP 24420 Pakistan; 2 Department of Mathematics, College of Science, Al-Zulfi, Majmaah University, Majmaah, 11952 Saudi Arabia; 3 Department of Mathematics, College of Science and Humanities in Al-Aflaj, Prince Sattam bin Abdulaziz University, Al-Kharj, Al-Aflaj 11942 Saudi Arabia; 4 Administration Department, Administrative Science College, Thamar University, Thamar, Yemen; 5 Petroleum Engineering Department, Faculty of Engineering and Technology, Future University in Egypt, New Cairo, 11835 Egypt

**Keywords:** Mathematics and computing, Physics

## Abstract

The present investigation focuses on the characteristics of heat and mass transfer in the context of their applications. There has been a lot of interest in the use of non-Newtonian fluids in engineering and biological disciplines. Having such considerable attention to non-Newtonian fluids, the goal is to explore the flow of Jeffrey non-Newtonian convective fluid driven by a non-linear stretching surface considering the effect of nonlinear chemical reaction effect. The relevant set of difference equations was changed to ordinary equations by using a transformation matrix. To create numerical solutions for velocity and concentration fields, the Runge–Kutta fourth-order method along with the shooting approach is utilized. The innovative fragment of the present study is to scrutinize the magnetized viscous non-Newtonian fluid over extending sheet with internal heat transfer regarding the inspiration of nonlinear chemical reaction effect, which still not has been elaborated on in the available works to date. Consequently, in the restrictive sense, the existing work is associated with available work and originated in exceptional agreement. Graphs depict the effects of various variables on motion and concentration fields, like the Hartman number, Schmidt number, and chemical reaction parameter. The performance of chemical reaction factor, Schmidt number, Hartmann number, and Deborah numbers on velocities component, temperature, and concentration profiles are discussed through graphs. The effect of emerging parameters in the mass transfer is also investigated numerically and 3D configuration is also provided. It is observed that the Deborah numbers and Hartmann numbers have the same effect on velocity components. Also, the thickness of the boundary layer reduces as the Hartmann number increases. As the Schmit number grows, the concentration field decline. For destructive and generative chemical reactions, the concentration fields observed opposite effects. It is also noticed that the surface mas transfer reduces as the Hartmann number rises. The statistical findings of the heat-transfer rate are also documented and scrutinized.

## Introduction

Non-Newtonian materials have received a lot of attraction in recent decades due to technical and materials science. Non-Newtonian activity can be observed in coating sheets, foodstuffs, fiber optics, digging muds, and thermoplastic polymers production processes. The Jeffrey liquid is one of the fluid models that have received a lot of interest from researchers. Relaxing and delay reactions may occur with this flow model. Extrusion operations, crystal and sheet manufacturing, semiconductor circuits, crystal growth, and other fields all benefit from studying boundary layer movements over a stretching cylinder. The boundary layer movement was introduced by Sakiadis^1^ beyond a continuous solid substrate flowing at a consistent rate. Many researchers expanded on Sakiadis' theory for stretching sheet moments in viscoelastic and non-Newtonian liquids heat fluxes under variable circumstances. The articles^2–10^ reflect some of the most recent work on the subject. Flows induced by a vertical stretched surface are considered in almost all of the research listed above. Some studies^11–15^ may also be addressed in these aspects. Raptis and Perdikis^16^ focused at magnetized viscous flow across a stochastic extending surface in the occurrence of a chemical process recently. The goal of this research is to (1) expand flow characteristics to Jeffrey liquids^16^ and (2) propose an numerical solution for a nonlinear dynamic situation.

For the numerical solution, a newly developed methodology called the Killer-box-method^17^ was used. Similar issues have been effectively solved using this strategy^18,19,19,19–30^.

The transport of convective heat can be improved by modifying the flow shape, the operating conditions, or by increasing the thermal sensitivity. For example, adding tiny particles with high electrical stability to a fluid improves its overall thermal properties. The microscopic droplets are referred to as nanostructures, and the solvent with no additives is referred to as conventional fluids, which includes water, methane, kerosene, and so on. There are two methods for depositing these nanoparticles; the first one is known as the single-step approach^31^ via direct vaporization. The second process, known as the two-step technique generated nanoparticles individually and subsequently disseminated them in the conventional fluids. Many studies have been conducted to study the characteristics that influence the nanoparticle’s resistance to heat flux. Unlike microbes, nanomaterial’s are not conscience; their movement is driven by Brownian motion and thermophoresis. The thicker microbes sink into the coolant, and indeed the microbes float to the surface. This replenishment causes a macroscopic movement known as bioconvection. Many studies^32^ purported to show the emergence of motile bacteria in nanofluid momentum. The heat transmission problem was examined employing Fourier law, but proved being too difficult to observe. Subsequently, with the assistance of Cattaneo and, later Christov^33–37^, a refined Fourier law was devised and applied to define and evaluate the heat transmission problem beyond extending cylinders.

Turkyilmazoglu^38^ investigated the longitudinal fins of rectangular profile past a stretching sheet and examined the heat transfer phenomena. The heat-mass transfer of magnetofydrodynamic flow over a permeable rotating sheet with variable viscosity was examined by Turkyilmazoglu^39^. Turkyilmazoglu^40^ studied the convection MHD flow with porous matrix and heat source. A closed form of solutions has been obtained for the thermal radiation. Similarly, Turkyilmazoglu^41^ investigated the dust Nano sized particles past a stretchable rotating sheet with two-phase heat analysis. The nonlinear solution has been obtained and discussed the convergence of adomian decomposition method by Turkyilmazoglu^41^. The heat transfer analysis with variable Prandtl numbers over a rotating disk of MHD flow with uniform electric field was explored by Turkyilmazoglu^42^. The hybrid nanofluid fluid with thermal radiation over a porous stretchable sheet with partial slip was examined by Turkyilmazoglu et al.^43^.

The innovative fragment of the present study is to scrutinize the magnetized viscous non-Newtonian fluid over extending sheet with internal heat transfer regarding the inspiration of variable viscosity and multiple slip effect, still not has been elaborated in the available works to date. Consequently, in the restrictive sense, the existing work is associated with available work and originated in exceptional agreement. It is observed that the Deborah numbers and Hartmann number have same effect on velocity components. Also the thickness of the boundary layer reduces as the Hartmann number increases. As the Schmidt number grows, the concentration field decline. For destructive and generative chemical reactions, the concentration fields observed opposite effects. It is also noticed that the surface mas transfer reduces as Hartmann number rising. The statistical findings of the heat-transfer rate are also documented and scrutinized.

The goal of the present study is to investigate the characteristics of heat and mass transfer in the context of their applications. There has been a lot of interest in the use of non-Newtonian fluids in engineering and biological disciplines. Having such a considerable attention in non-Newtonian fluids, the goal is to explore the flow of Jeffrey non-Newtonian mixed convective fluid driven by a non-linear permeable rotating stretching surface considering with suction or injection and nonlinear thermal radiation effect. The relevant set of difference equations was changed to ordinary equations by using a transformation matrix. To create numerical solutions for velocity and concentration fields, the Runge–Kutta fourth-order method along with the shooting approach is utilized.

The following is the outline of the presentation. The problem's concept is presented in “ Mathematical modelling” section. “Numerical procedure and validation of code” section deduces velocity and concentration numerical solutions. The consolidation of the resulting solutions is explicitly examined in “Results and discussion” section. The explanation of graphical form is covered in “Closing remarks” section. Section 6 summarises the findings.

## Mathematical modelling

**Figure 1:**
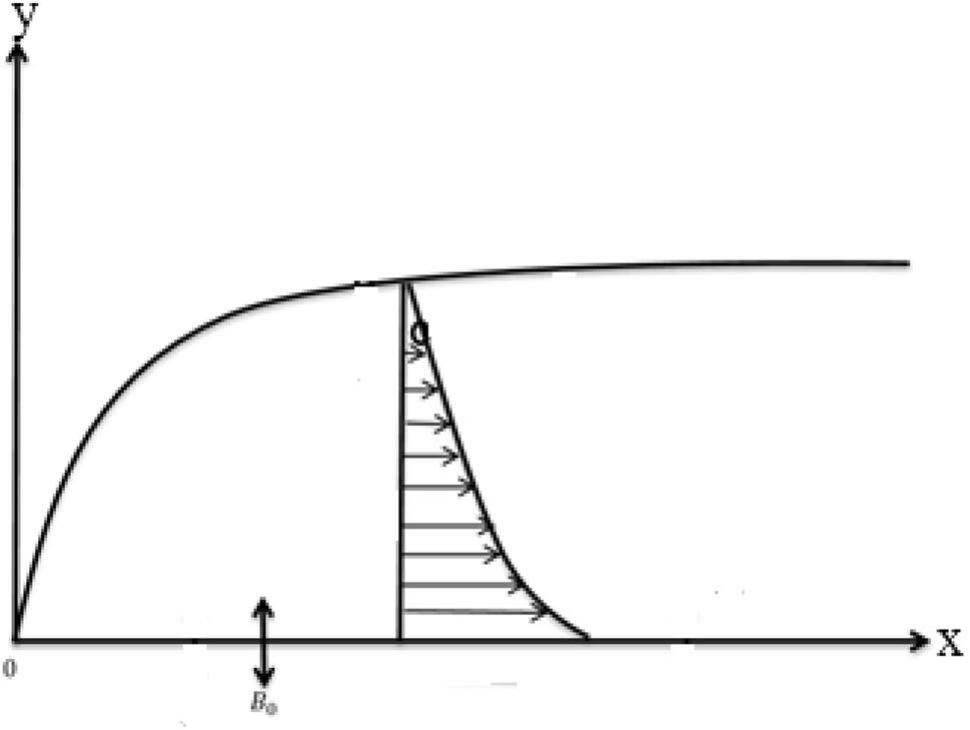
Geometry of the mathematical modelling.

Figure 1 shows the geometry of the problem. A 2D Jeffrey liquid that flows across a magnetized stretched surface is considered. It is assumed that the x-axis is along the stretch sheet, while the y-axis is vertical. In the y-direction, a homogeneous magnetic flux exerts its influence. The resultant magnetic force is ignored for tiny magnetic Reynolds numbers. We also took into account the possibility of a first-order reaction.

The equations that regulate the stream in this situation are as follows^9,12,14^:

1 $$ \frac{\partial u}{{\partial x}} + \frac{\partial v}{{\partial y}} = 0 $$

2 $$ \begin{aligned} u\frac{\partial u}{{\partial x}} + v\frac{\partial u}{{\partial y}} & = \lambda_{1} \left( {u^{2} \frac{{\partial^{2} u}}{{\partial x^{2} }} + v^{2} \frac{{\partial^{2} u}}{{\partial y^{2} }} + 2uv\frac{{\partial^{2} u}}{\partial y\partial x}} \right) \\ & = \nu \left( {\frac{{\partial^{2} u}}{{\partial y^{2} }} + \lambda_{2} \left( {u\frac{{\partial^{2} u}}{{\partial y^{2} \partial x}} + v\frac{{\partial^{3} u}}{{\partial y^{3} }} - \frac{\partial u}{{\partial x}}\frac{{\partial^{2} u}}{{\partial y^{2} }} - \frac{\partial u}{{\partial x}}\frac{{\partial^{2} u}}{{\partial y^{2} }}} \right)} \right) - \frac{{\sigma B_{0}^{2} }}{\rho } \\ \end{aligned} $$

3 $$ u\frac{\partial T}{{\partial x}} + v\frac{\partial T}{{\partial y}} = D\frac{{\partial^{2} T}}{{\partial y^{2} }} - RT $$

In the upstairs balances, $u$ and $v$ are velocities lengthwise *x* and *y*-directions, individually, $\nu$ the kinematic viscosity, $\rho$ the density, $\sigma$ the conductivity, $\lambda_{1} ,\lambda_{2}$ the relaxation/retardation time, $T$ the concentration, $D$ the diffusion quantity, and $R$ is the reaction factor.

The suitable constraints are^9,10^

4 $$ \begin{aligned} & u(x,y) = a_{1} x + b_{1} x^{2} \\ & v(x,y) = 0,T(x,y) = T_{w} \, at \, y = 0 \\ & u \to 0,T \to 0 \, as \, y \to \infty \\ \end{aligned} $$

here $a_{1} {\text{ and }}b_{1}$ are assumed to be constants.

For simplicity of the problem, we familiarize the following similarities:

5 $$ \begin{aligned} & \eta { = }\left( {\tfrac{{a_{1} }}{\nu }} \right)^{\frac{1}{2}} y,u = a_{1} xf^{^{\prime}} \left( \eta \right) + b_{1} x^{2} g^{^{\prime}} \left( \eta \right),v = - \left( {a_{1} v} \right)^{\frac{1}{2}} f\left( \eta \right) - 2b_{1} x\left( {\tfrac{\nu }{{a_{1} }}} \right)^{\frac{1}{2}} g\left( \eta \right), \\ & T = T_{w} \left( {T_{0} \left( \eta \right) + \frac{{2b_{1} x}}{{a_{1} }}T_{1} \left( \eta \right)} \right) \\ \end{aligned} $$

here prime signifies the derivative with reverence to η. Equation 1 is pleased automatically and Eqs. (2–4) are distorted as monitors:

6 $$ \begin{aligned} & f^{\prime \prime \prime } (\eta ) + M^{2} f^{\prime } (\eta ) - f^{\prime } (\eta )^{2} + f(\eta )f^{\prime \prime } (\eta ) + \Omega_{1} \left( {2f(\eta )f^{\prime } (\eta )f^{\prime \prime } (\eta ) - f(\eta )^{2} f^{\prime \prime \prime } (\eta )} \right) \\ & + \Omega_{2} \left( {f^{\prime \prime } (\eta )^{2} - f(\eta )f^{\prime \prime \prime \prime } (\eta )} \right) = 0 \\ \end{aligned} $$

7 $$ \begin{aligned} & g^{\prime \prime \prime } (\eta ) - M^{2} g^{\prime } (\eta ) - 3f^{\prime } (\eta )g^{\prime } (\eta ) + 2g(\eta )f^{\prime \prime } (\eta ) + f^{\prime } (\eta )g^{\prime \prime } (\eta ) \\ & + \Omega_{1} \left( \begin{gathered} 4f(\eta )f^{\prime } (\eta )g^{\prime \prime } (\eta ) + 2f(\eta )g^{\prime } (\eta )f^{\prime \prime } (\eta ) - f(\eta )^{2} g^{\prime \prime \prime } (\eta ) - 4f(\eta )g(\eta )f^{\prime \prime \prime } (\eta ) \hfill \\ + 4f^{\prime } (\eta )g(\eta )f^{\prime \prime } (\eta ) - 2f^{\prime } (\eta )^{2} g^{\prime } (\eta ) \hfill \\ \end{gathered} \right) \\ & + \Omega_{2} \left( {f^{\prime } (\eta )g^{\prime \prime \prime } (\eta ) - g^{\prime } (\eta )f^{\prime \prime \prime } (\eta ) - f(\eta )g^{\prime \prime \prime \prime } (\eta ) - 2g(\eta )f^{\prime \prime \prime \prime } (\eta ) + 3f^{\prime \prime } (\eta )g^{\prime \prime } (\eta )} \right) = 0 \\ \end{aligned} $$

8 $$ T_{0}^{\prime \prime } + ScfT_{0}^{\prime } - Sc\Upsilon T_{0} = 0 $$

9 $$ T_{1}^{\prime \prime } + Scf(\eta )T_{1}^{\prime } - Scf^{\prime } (\eta )T_{1} - Sc\Upsilon T_{1} + Scg(\eta )T_{0}^{\prime } = 0 $$

10 $$ \begin{aligned} & f = 0,f^{\prime } = 1,g = 0,g^{\prime } = 1,T_{0} = 1,T_{1} = 0;{\text{ at }}\eta \to 0 \\ & f^{\prime } = 0,g^{\prime } = 0,T_{0} = 0,T_{1} = 0;{\text{ at }}\eta \to \infty \\ \end{aligned} $$

In above equations Υ denotes chemical reaction factor, Sc stands for Schmidt number, M is the Hartman number, and Ω_1_ ,Ω_2_ are the Deborah numbers which are defiend as

11 $$ \Upsilon = \frac{R}{{a_{1} }},S = \frac{\nu }{D},M_{1}^{2} = \frac{{\sigma B_{0}^{2} }}{{a_{1} \rho }},\Omega_{1} = \lambda_{1} a_{1} ,\Omega_{2} = \lambda_{2} a_{1} . $$

The expressions of the mass transfer $T_{0}$ and $T_{1}$ at the wall are

12 $$ \begin{gathered} T_{0}^{\prime } \left( 0 \right) = \left( {\frac{{\partial T_{0} }}{\partial \eta }} \right)_{\eta = 0} \le 0, \hfill \\ T_{1}^{\prime } \left( 0 \right) = \left( {\frac{{\partial T_{1} }}{\partial \eta }} \right)_{\eta = 0} \le 0, \hfill \\ \end{gathered} $$

## Numerical procedure and validation of code

**Figure 2:**
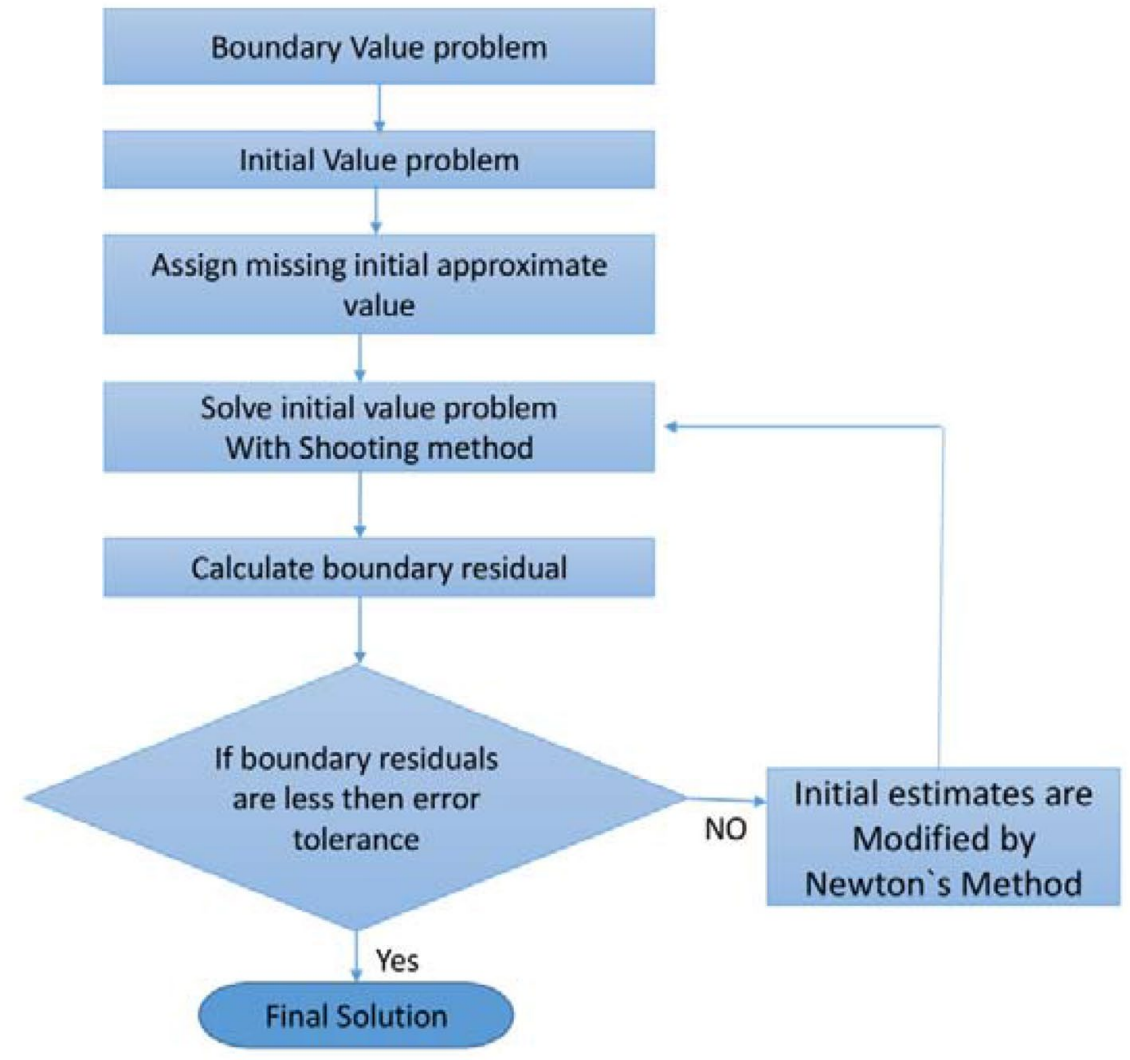
Flow chart of the numerical scheme.

**Figure 3:**
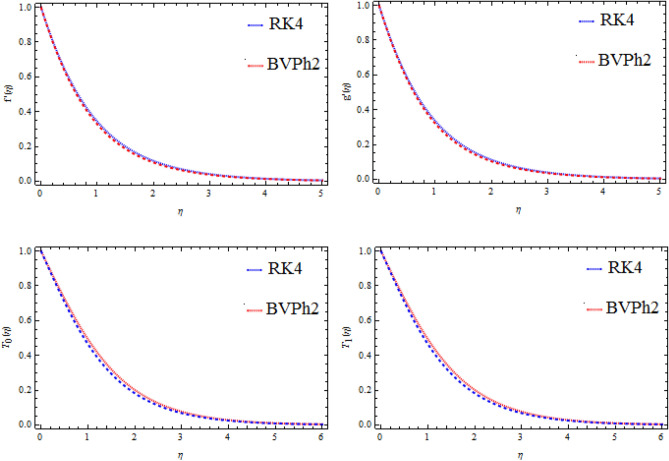
Comparison of RK4 and BVPh2, for velocities $f^{\prime}{\text{ and }}g^{\prime}$ and mass transfer $T_{0}$ and $T_{1}$.

The scheme of Eqs. (6–10) are treated numerically by Runge–Kutta 4th order-scheme. For this goal the governing equivalences are transformed to the conventional differential balances by the resemblance conversion. The transformed set of equations is further altered into order-first differential equations. The relevant constraints are also transformed to the initial constraints. Applying the numerical method (RK4), the transformed dimensionless system of equations is calculated using step size Δ$\upeta =0.01$. The current study is associated with published (see Table 1). The repetition practice is clogged up to meeting situations 10^−5^. For endorsement of the consequences, the BVPh2 is also functional and admirable settlement is established as revealed in Fig. 2. The procedure of the numerical method is given in Fig. 3.

**Table 1:** Comparison of the variation of surface mass transfer $- T_{0}^{\prime } (0)$ and $- T_{1}^{\prime } (0)$ for some values of $Sc$, $\Upsilon$, $M$ $\Omega_{1} = \Omega_{2}$ with published work^21^.

$Sc$	$\Upsilon$	$M$	$\Omega_{1} = \Omega_{2}$	$- T_{0}^{\prime } (0)$	$- T_{1}^{\prime } (0)$	Liao^21^ $- T_{0}^{\prime } (0)$	Liao^21^ $- T_{1}^{\prime } (0)$
1	0.3	0.5	0.3	0.7230	0.1453	0.7231	0.1451
	0.7			0.8471	0.1414	0.8472	0.1412
	1.2			1.0447	0.1352	1.0443	0.1354
	1.8			1.3261	0.1140	1.3260	0.1146
0.4	2.2			0.9832	0.0291	0.9831	0.0299
0.8				0.9982	0.0954	0.9984	0.0951
1.2				1.3164	0.1543	1.3161	0.1546
1.6				1.5514	0.1950	1.5519	0.1952
2.2	1.3	0		1.2664	0.1347	1.2667	0.1347
		0.2		1.2542	0.1368	1.2543	0.1367
		0.4		1.1320	0.1377	1.1321	0.1376
		0.8		1.1142	0.1386	1.1142	0.1380
		1.0		1.0531	0.1399	1.0532	0.1393
		1.8	0	1.2537	0.1235	1.2536	0.1237
			0.3	1.2680	0.1343	1.2683	0.1348
			0.9	1.2884	0.1352	1.2887	0.1351
			1.2	1.2910	0.1367	1.2912	0.1360
			1.5	1.2997	0.1380	1.2991	0.1382

## Results and discussion

In this work we have investigated the mixed convection flow of a Jeffrey non-Newtonian fluid over a stretchable surface. The problem is modelled and then solved numerically using the Runge–Kutta fourth-order method together with the shooting technique. The physical model of the problem is shown in Fig. 1. Numerical results are computed in terms of plots and table. Figures 2 and 3, are discussed in “Numerical procedure and validation of code” section. These plots depict the effects of various variables on motion and concentration fields, like the Hartman number, Schmidt number, and chemical reaction parameter. On $f^{\prime } {\text{ and }}g^{\prime }$, the performance of $\Omega_{1}$ and $M_{1}$ is same. The thickness of the boundary layer is reduced when the value of $M_{1}$ is increased. As Sc grows, the concentration fields $T_{0}$ and $T_{1}$ decline. The destructive response ($\Upsilon$ > 0) has the effect of lowering the concentration profiles. For destructive ($\Upsilon$ > 0) and generative ($\Upsilon$ < 0) chemical reactions, the concentration fields $T_{0}$ and $T_{1}$ observed opposite effects. By raising $M_{1}$, the surface mass transfer reduces. The statistical findings of the heat-transfer rate are also documented and scrutinized.

The pictorial results for the influence of Deborah numbers $\Omega_{1} ,\Omega_{2}$ Hartman number $M$, Schmidt number $Sc$, and the chemical reaction factor $\Upsilon$ on $f^{\prime}{\text{ and }}g^{\prime}$ are shown in this section. Figures 4, 5, 6, 7, 8, 9, 10, 11, 12, 13, 14, 15, 16, 17, 18, 19, 20, 21, 22, 23, 24 and 25 provide examples of such impacts with physical amplifications.

**Figure 4:**
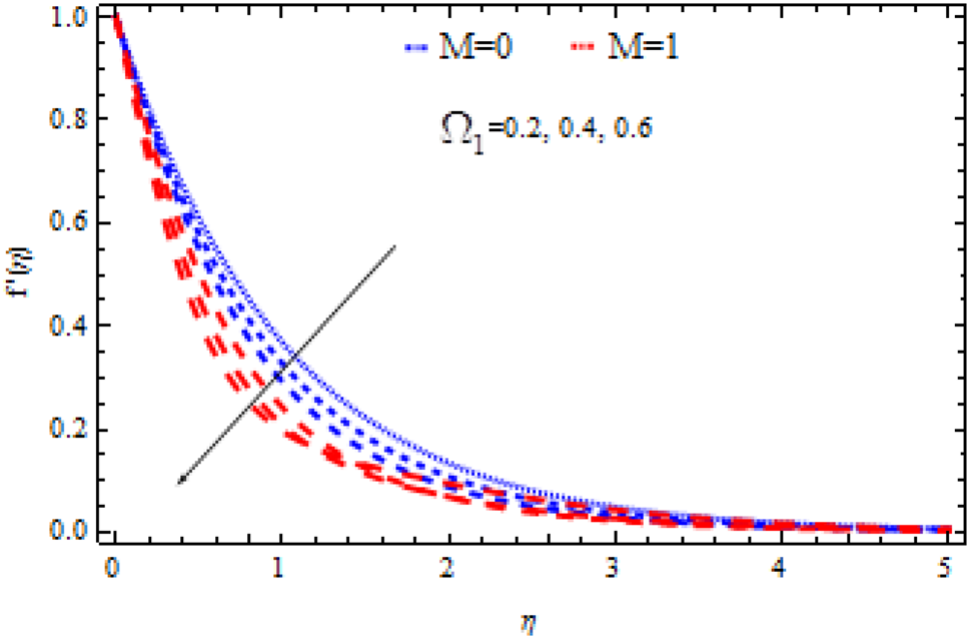
Consequence of $\Omega_{1}$ on $f^{\prime }$.

**Figure 5:**
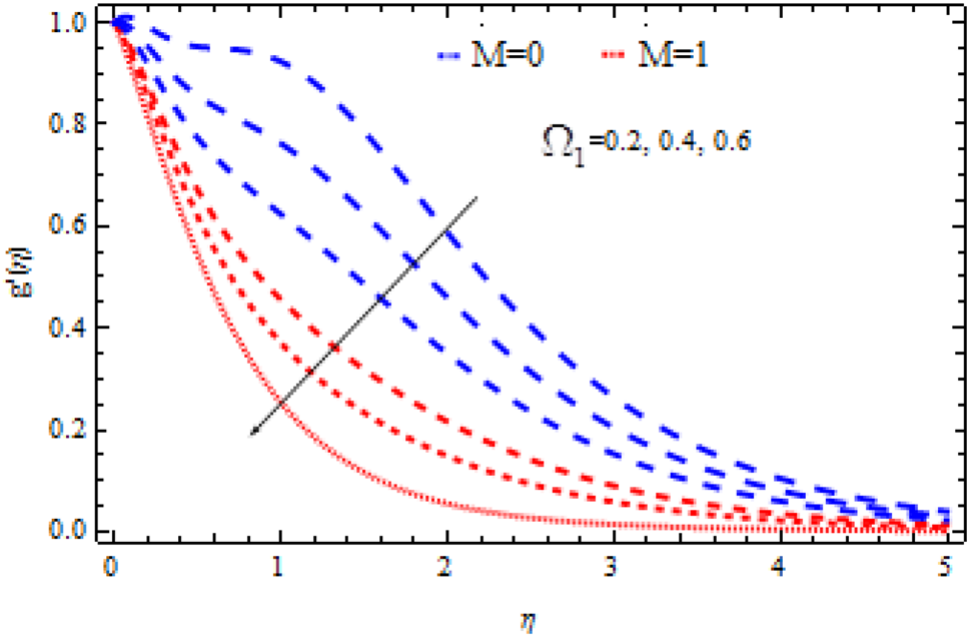
Consequence of $\Omega_{1}$ on $g^{\prime }$.

**Figure 6:**
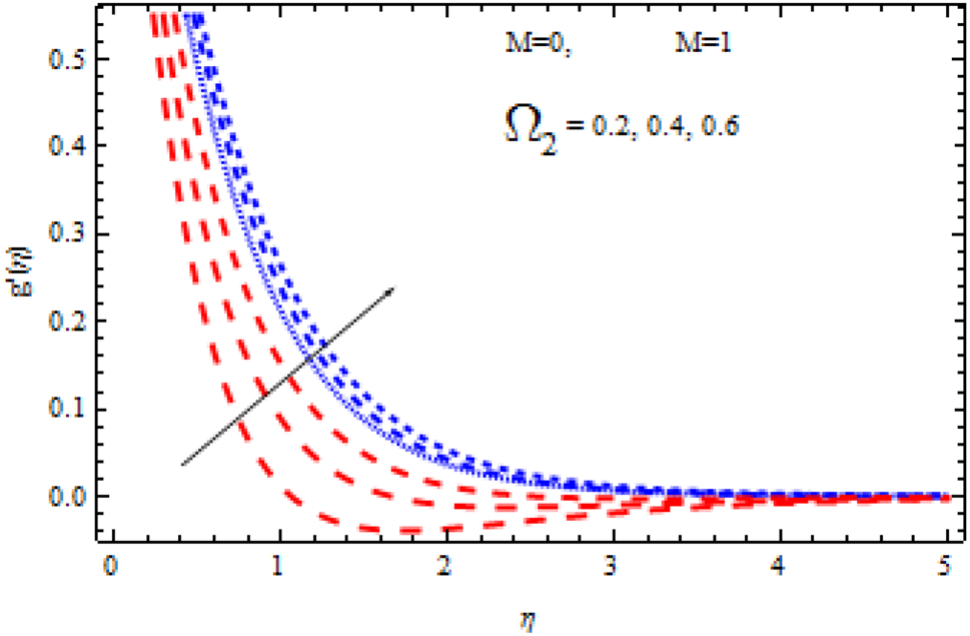
Consequence of $\Omega_{2}$ on $f^{\prime }$.

**Figure 7:**
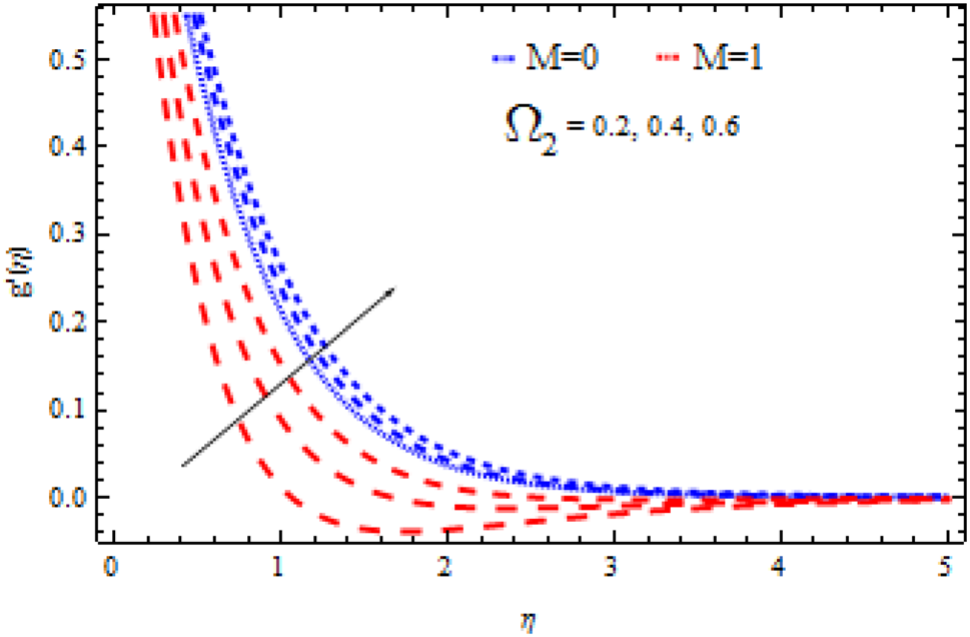
Consequence of $\Omega_{2}$ on $g^{\prime }$.

**Figure 8:**
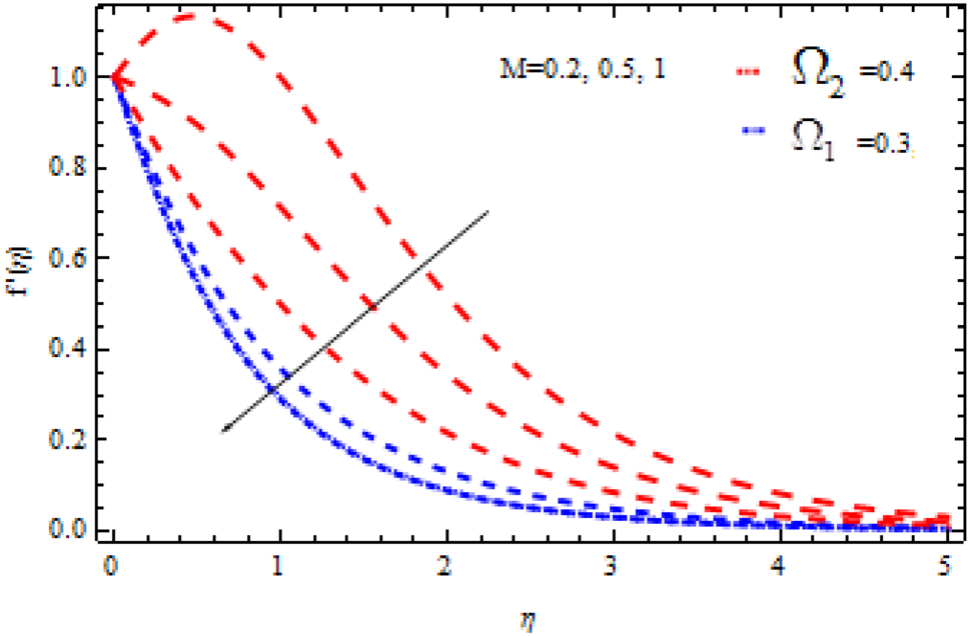
Consequence of $M$ on $f^{\prime }$.

**Figure 9:**
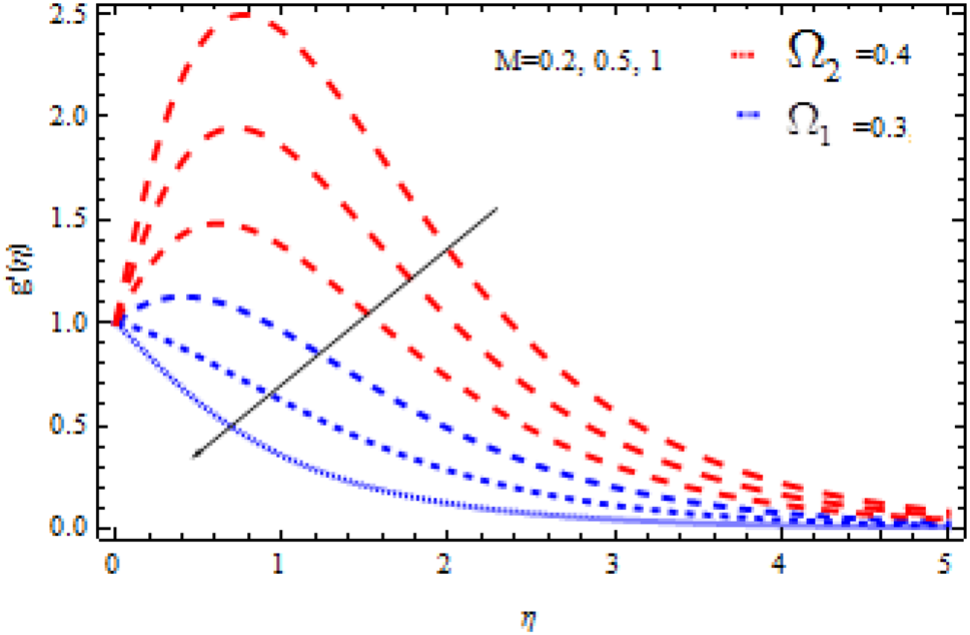
Consequence of $M$ on $g^{\prime }$.

**Figure 10:**
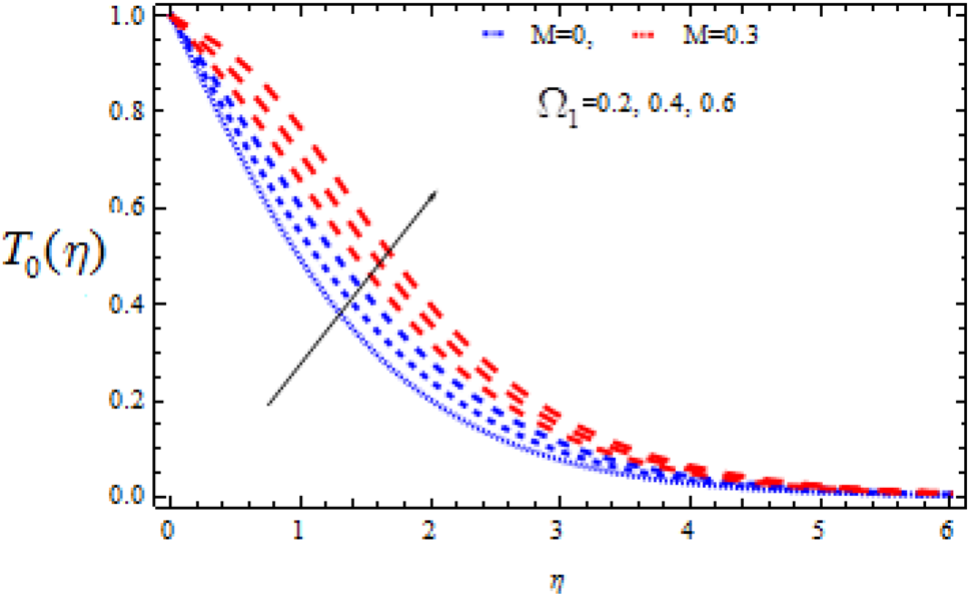
Consequence of $\Omega_{1}$ on $T_{0} (\eta )$.

**Figure 11:**
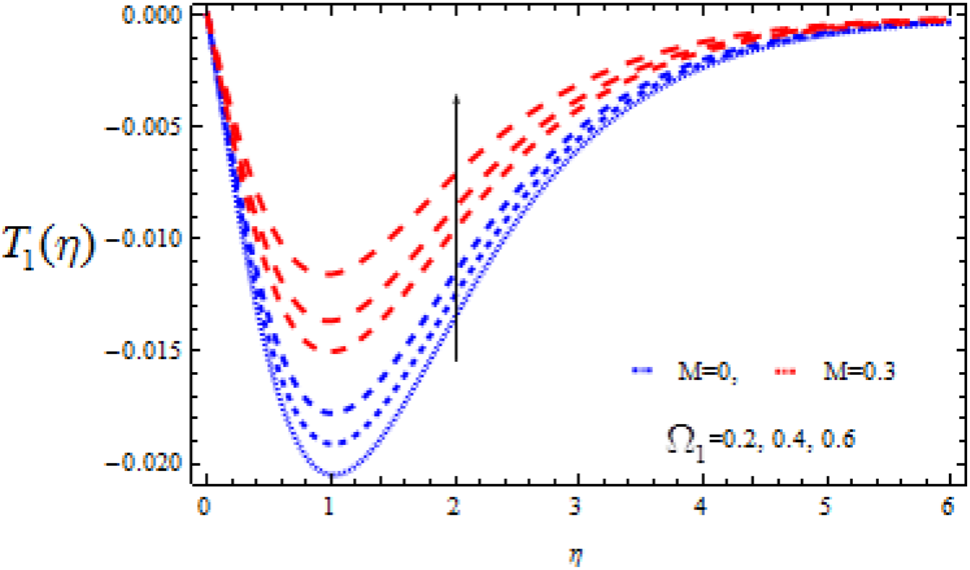
Consequence of $\Omega_{1}$ on $T_{1} (\eta )$.

**Figure 12:**
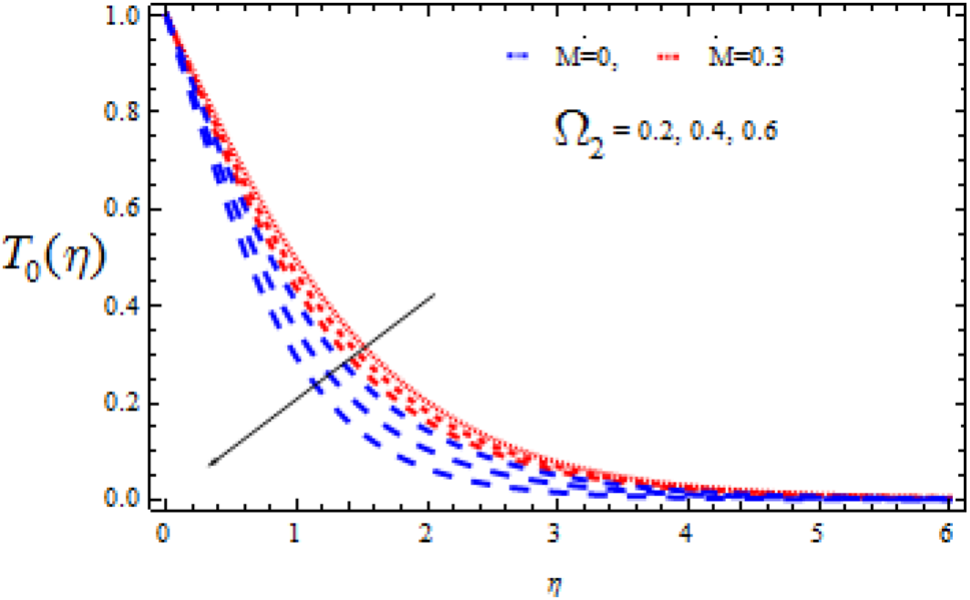
Consequence of $\Omega_{2}$ on $T_{0} (\eta )$.

**Figure 13:**
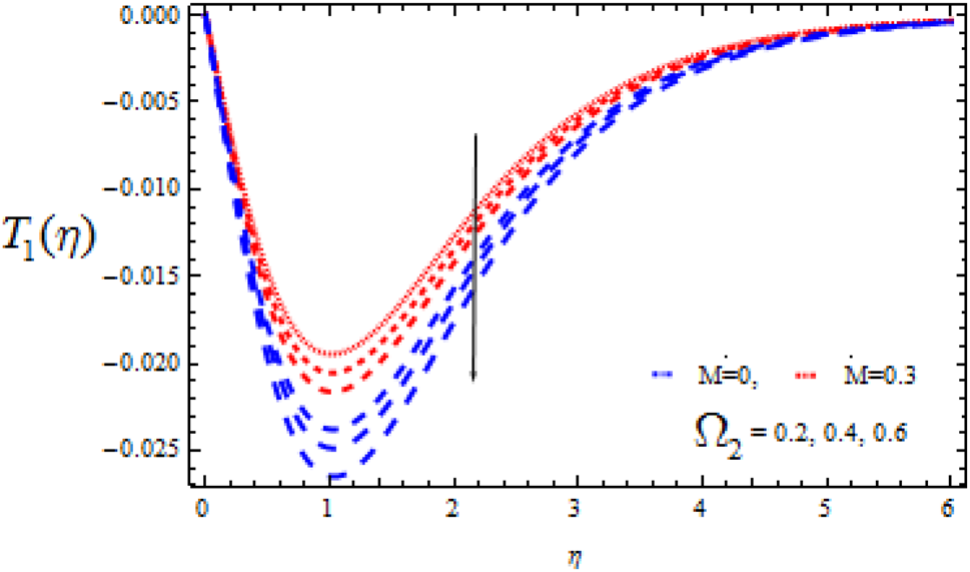
Consequence of $\Omega_{2}$ on $T_{1} (\eta )$.

**Figure 14:**
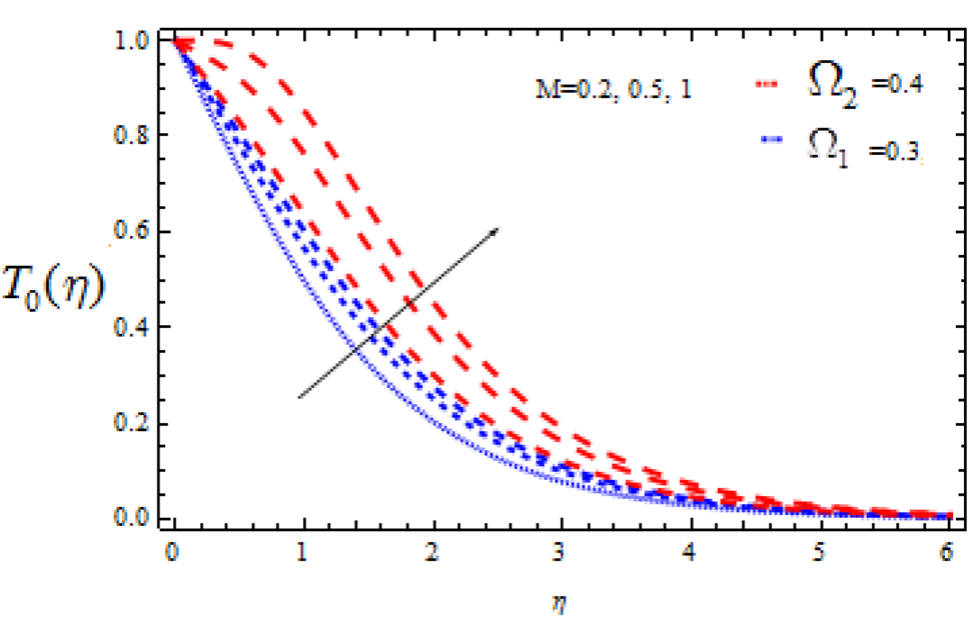
Consequence of $M$ on $T_{0} (\eta )$.

**Figure 15:**
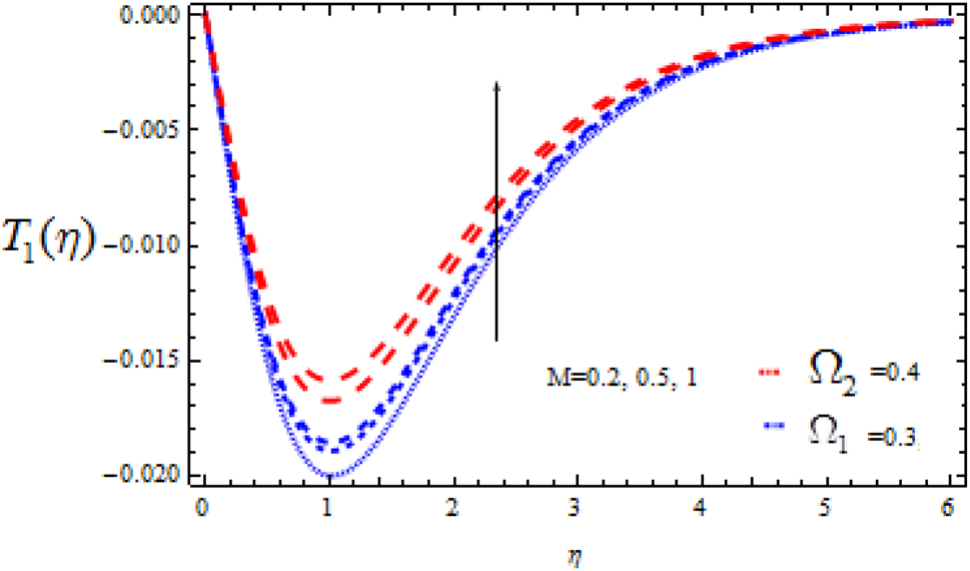
Consequence of $M$ on $T_{1} (\eta )$.

**Figure 16:**
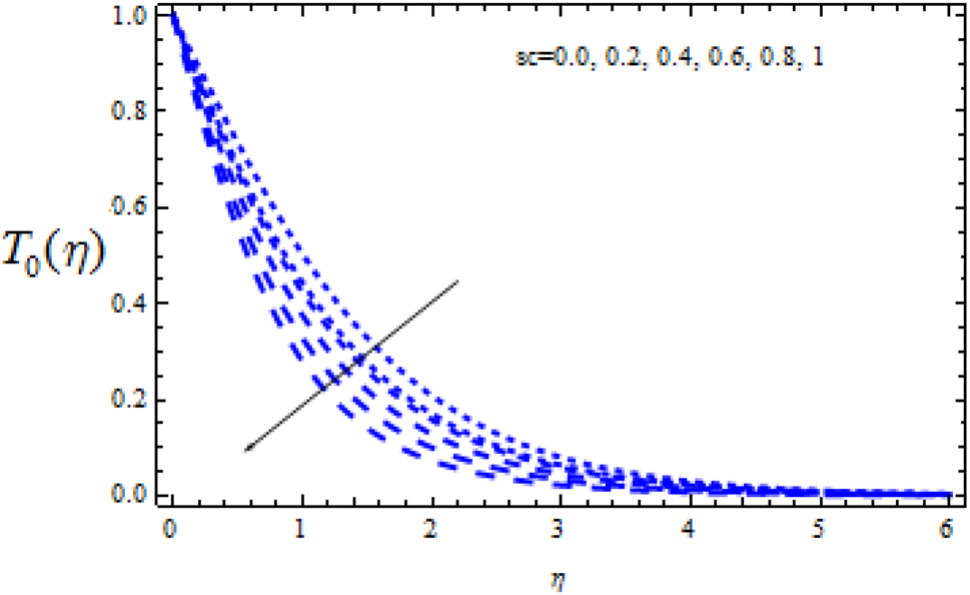
Consequence of $Sc$ on $T_{0} (\eta )$.

**Figure 17:**
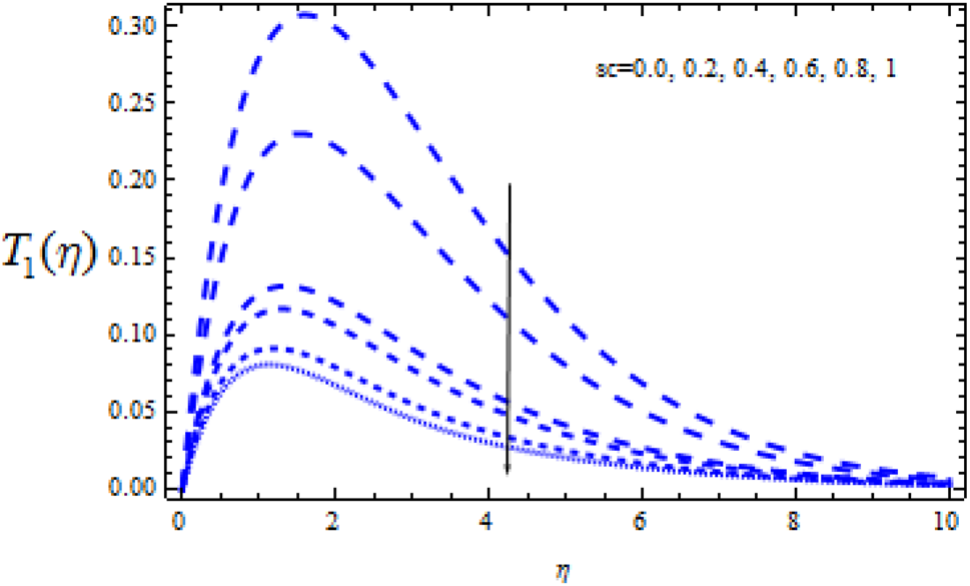
Consequence of $Sc$ on $T_{1} (\eta ). \,$.

**Figure 18:**
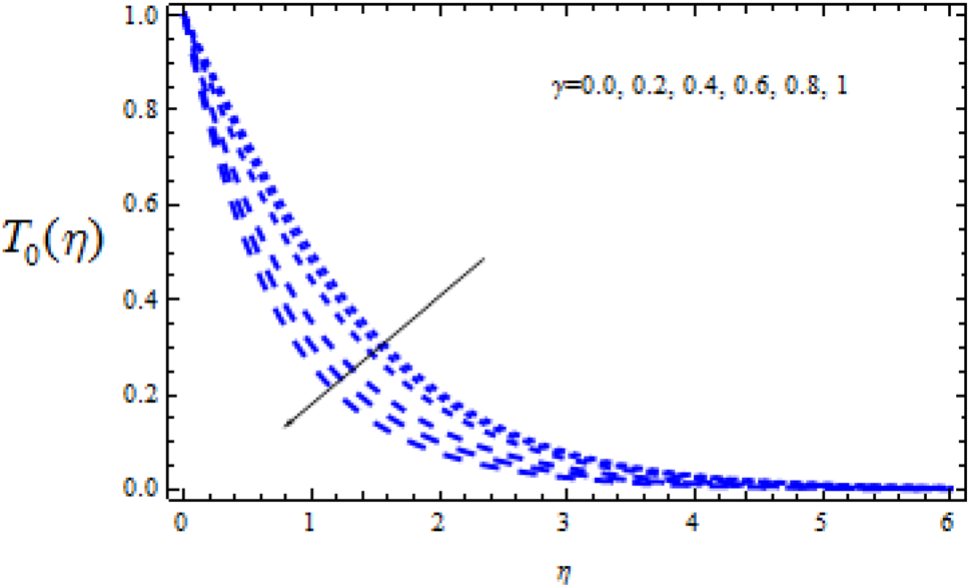
Consequence of $\Upsilon$ on $T_{0} (\eta )$.

**Figure 19:**
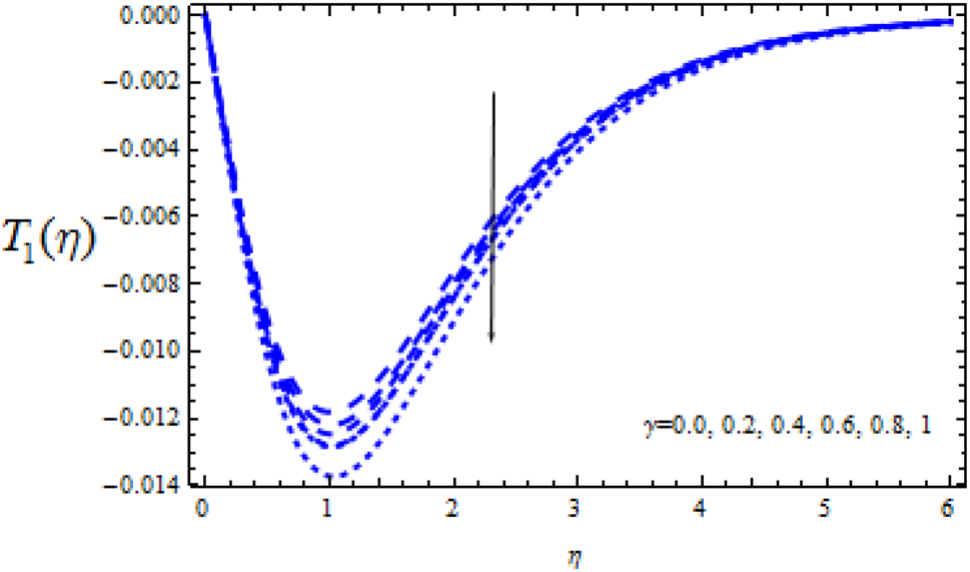
Consequence of $\Upsilon$ on $T_{1} (\eta )$.

**Figure 20:**
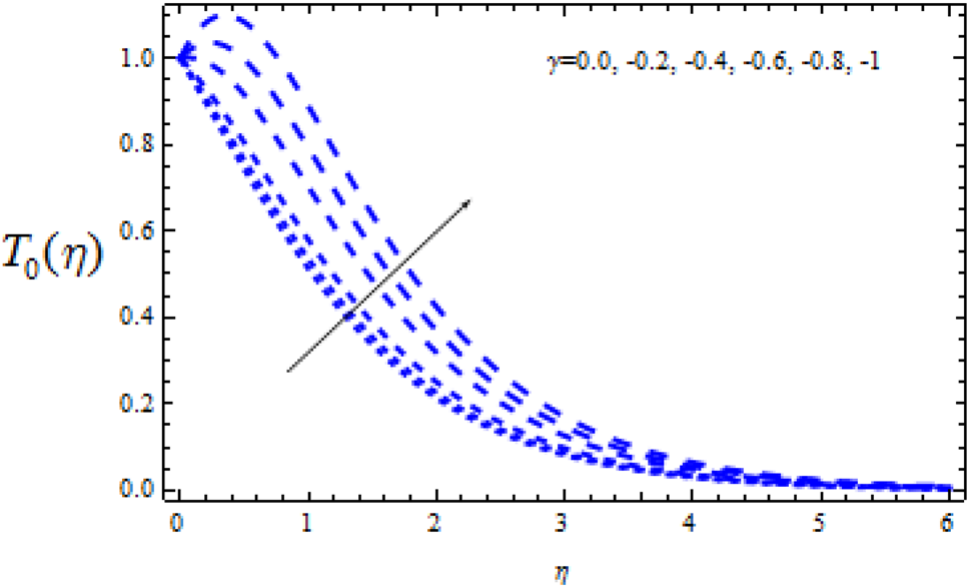
Consequence of $\Upsilon$ on $T_{0} (\eta )$.

**Figure 21:**
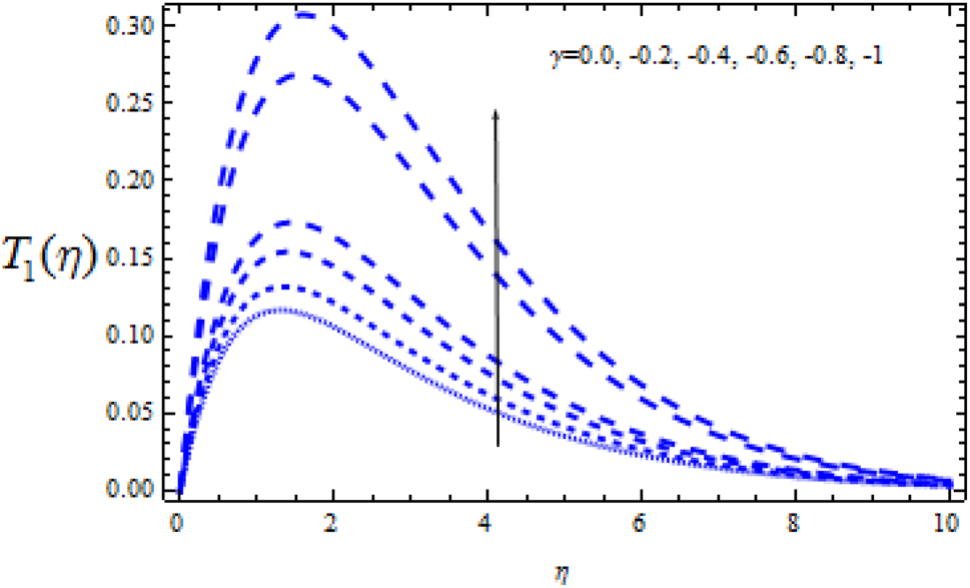
Consequence of $\Upsilon$ on $T_{1} (\eta )$.

**Figure 22:**
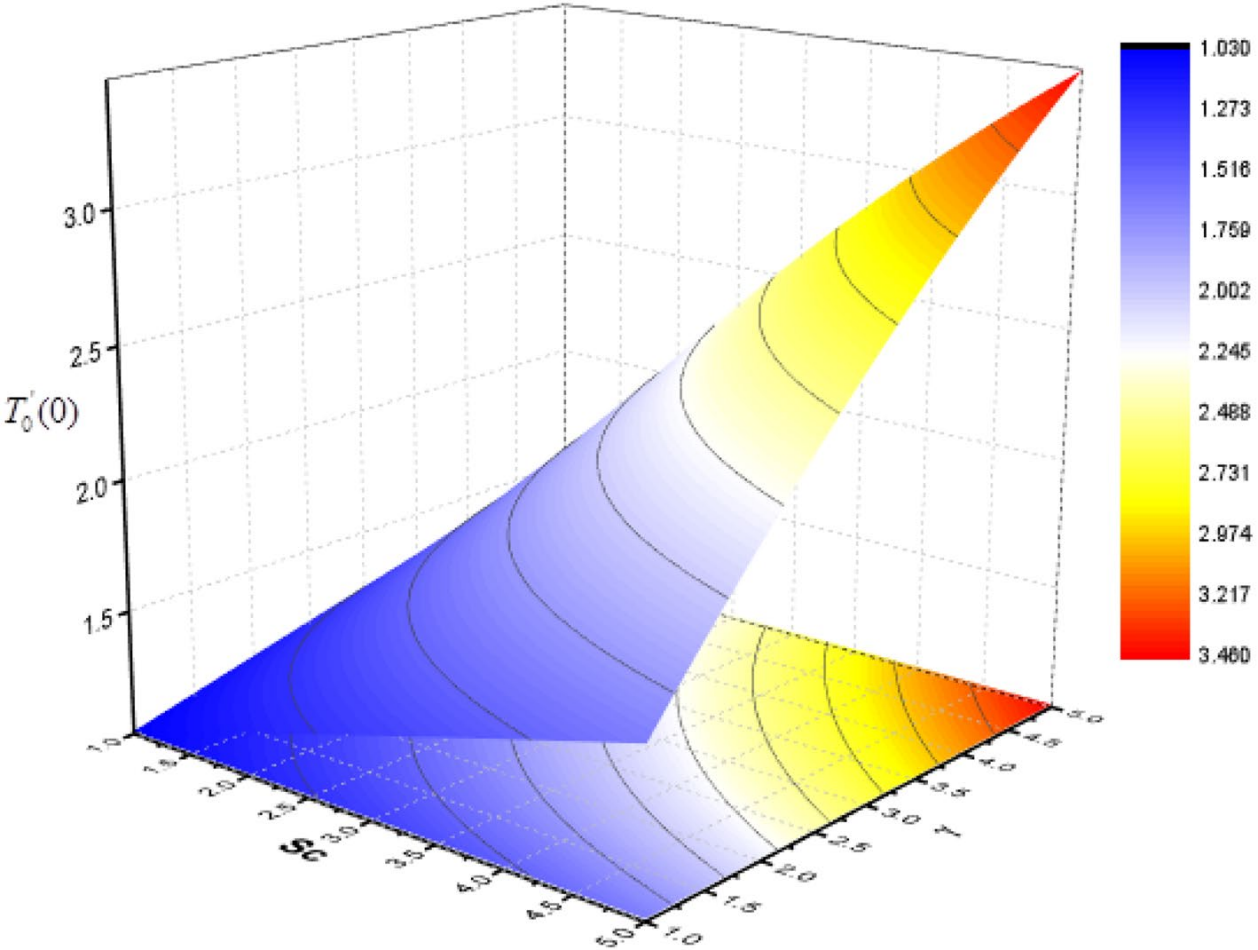
Consequence of $Sc{\text{ and }}\Upsilon$ on $T_{0}^{\prime } (0)$.

**Figure 23:**
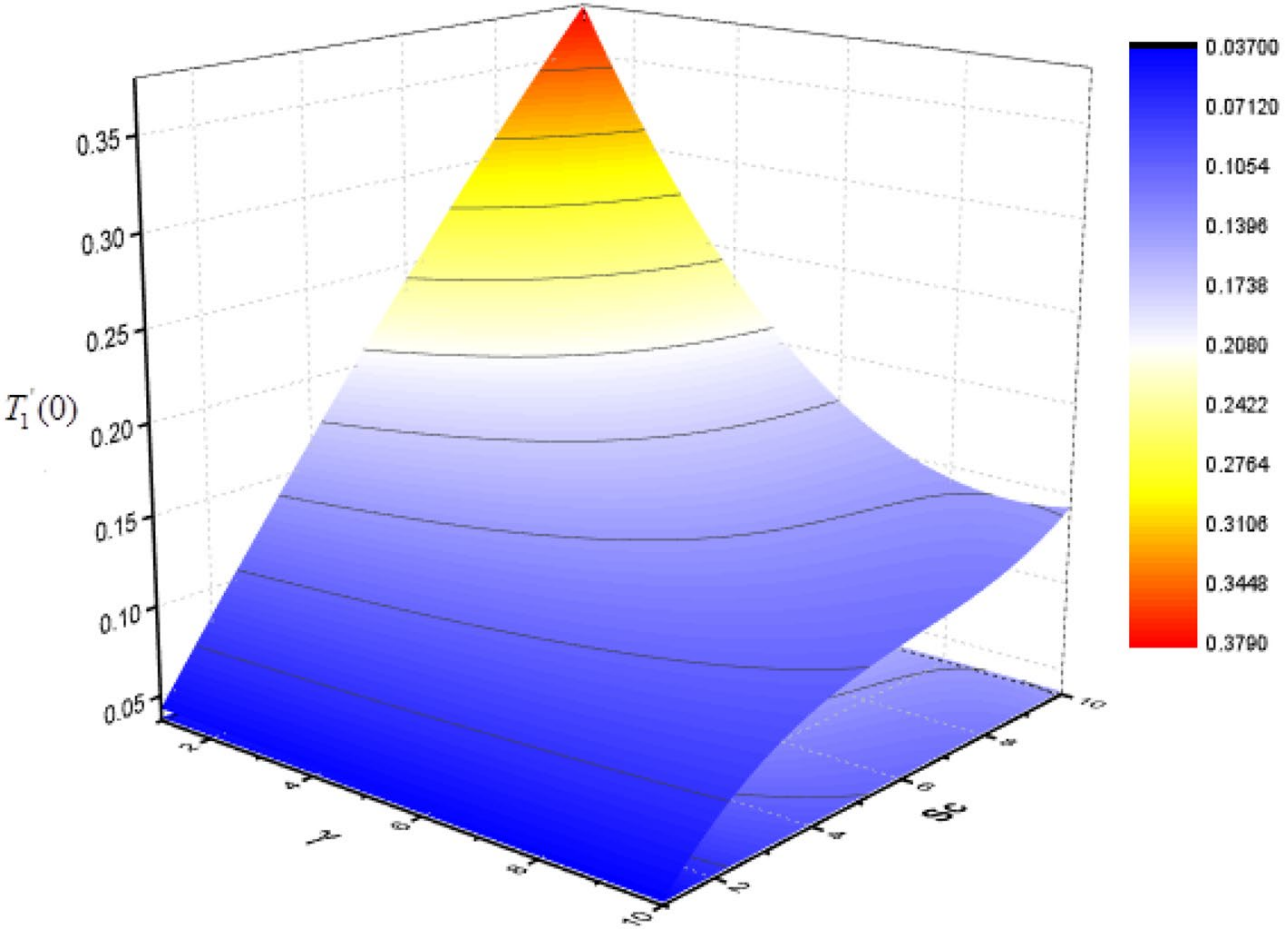
Consequence of $Sc{\text{ and }}\Upsilon$ on $T_{1}^{\prime } (0)$.

**Figure 24:**
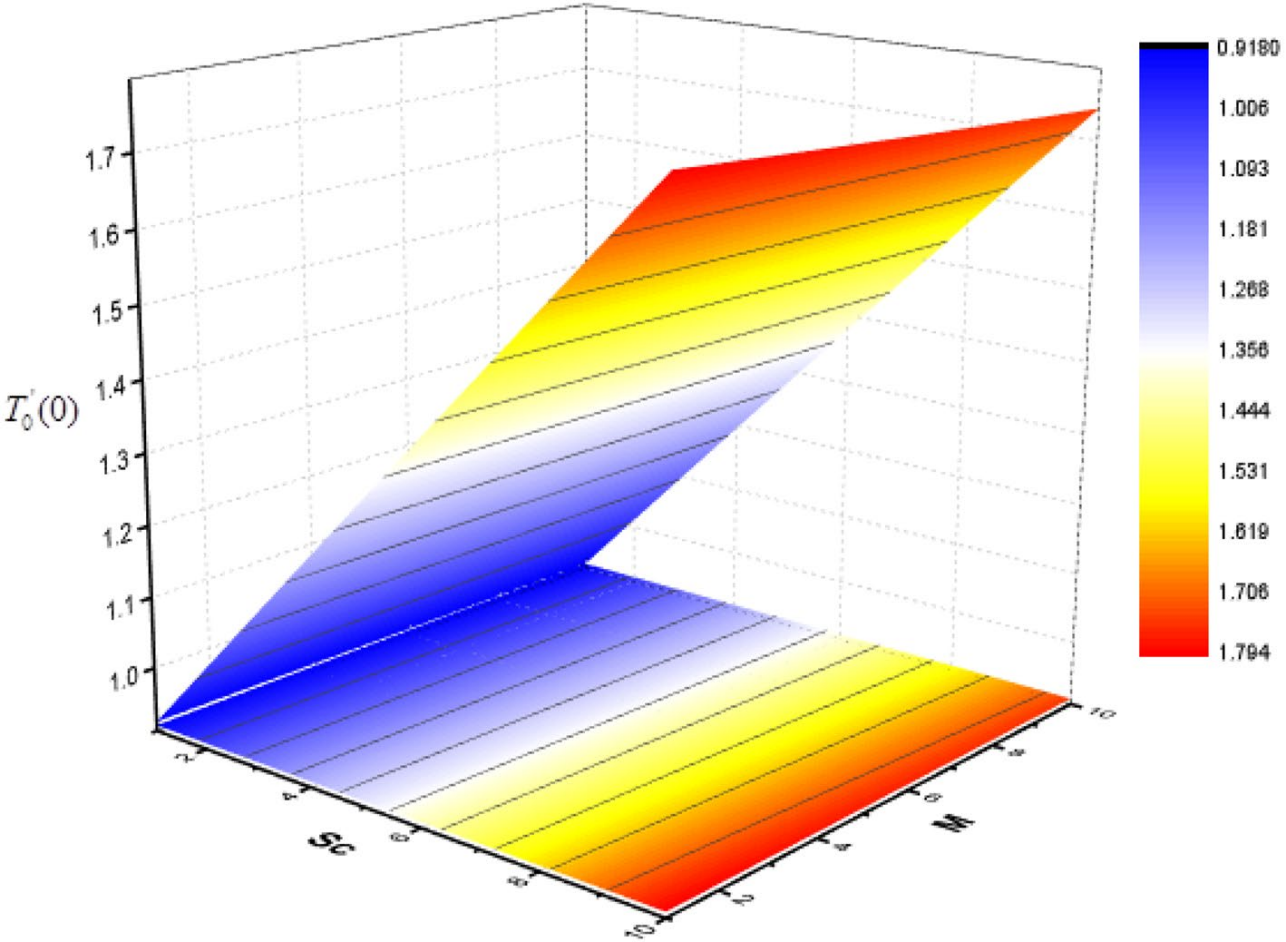
Consequence of *Sc* and *M* on $T_{0}^{\prime } (0)$.

**Figure 25:**
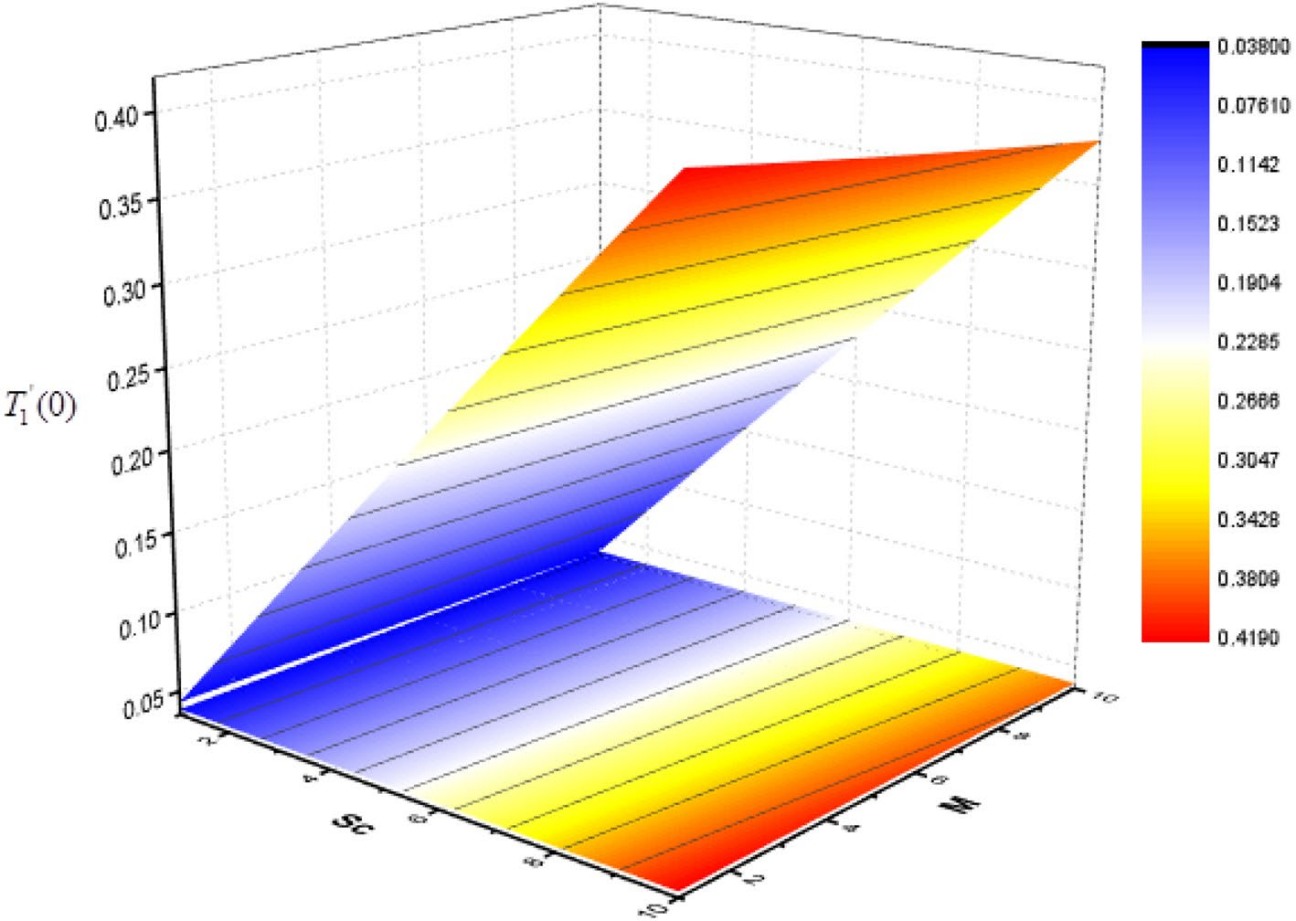
Consequence of *Sc* and *M* on $T_{1}^{\prime } (0)$.

The consequences of $\Omega_{1} ,\Omega_{2}$ and $M$ on $f^{\prime}{\text{ and }}g^{\prime}$ are examined in Figs. 4, 5, 6 and 7.

Figures 4 and 5 show the flow characteristics for varying parameter of Deborah number $\Omega_{1}$ in the presence and absence of M. It is detected that, $f^{\prime } {\text{ and }}g^{\prime }$ decline across the boundary layer with the growing values of $\Omega_{1}$. The velocity profiles $f^{\prime } {\text{ and }}g^{\prime }$ are shown to be decreased as $\Omega_{1}$ rises. When compared to $g^{\prime }$, however, the upsurge in $f^{\prime }$ is smaller. As Deborah number $\Omega_{1}$ is enhanced, the film thickness reduces. In contrast the Deborah number $\Omega_{2}$ has an opposite influence on the velocities $f^{\prime } {\text{ and }}g^{\prime }$ than $\Omega_{1}$ as shown in Figs. 6 and 7.

From these figures it is clear crystal the influence of $\Omega_{1}$ and $\Omega_{2}$ on $f^{\prime } {\text{ and }}g^{\prime }$ is higher in the absence of M. Figures 8 and 9 display that the inspiration of the Hartman number M on the velocities $f^{\prime } {\text{ and }}g^{\prime }$ in the presence and absence of Deborah number. Figure 8 displays the influence of M on the velocity component $f^{\prime }$ which is a function of $\eta$ . It is observed that the velocity reduces as the M raising. Also, from Fig. 9, it is quite evident that the velocity $g^{\prime }$ significantly decline as the M increases. The effect of M on $f^{\prime}{\text{ and }}g^{\prime}$ is analogous to that of $\Omega_{1}$. From this analysis it is also investigated that the effect of M for $\Omega_{2} = 0.4$ is higher than $\Omega_{2} = 0.3.$

Figures 10, 11, 12, 13, 14, 15, 16, 17, 18, 19, 20 and 21 show the fluctuations of the entrepreneurs on the concentration components $T_{0}$ and $T_{1}$. Figures 10 and 11 demonstrate the impact of Deborah number $\Omega_{1}$ on $T_{0}$ and $T_{1}$ in the event of a destructive chemical change ($\Upsilon$ > 0). Whenever the value of $\Omega_{1}$ is higher, the amplitude of the concentration profile $T_{0}$ grows, whereas the magnitude of $T_{1}$ drops. It should be noted that for high values of $\Omega_{1}$, the variance in $T_{1}$ is greater than that in $T_{0}$.

In the event of a destructive chemical reaction ($\Upsilon$ > 0), Figs. 12 and 13 explore the fluctuations of $\Omega_{2}$ on the concentration fields $T_{0}$ and $T_{1}$ for two different values of M = 0 and M = 0.3. When linking Figs. 10 and 11, it can be seen that Figs. 12 and 13 have the opposite qualitative consequences. Also, for M = 0, the concentration fields rapidly decreases as compare with M = 0.3.

The consequence of Hartmann number $M$ on $T_{0}$ and $T_{1}$ is seen in Figs. 14 and 15 respectively in the presence of Deborah numbers $\Omega_{1} = 0.3{\text{ and }}\Omega_{2} = 0.4.$ As $M$ is enhanced, the concentration fields $T_{0}$ and $T_{1}$ are found to increase. In both cases the enhancement is more significant for large value of Deborah number i.e., for $\Omega_{2} = 0.4$.

Figures 16 and 17 show how the Schmidt number Sc varies upon concentration profiles $T_{0}$ and $T_{1}$. When Sc grows, respectively,$T_{0}$ and $T_{1}$ drop. Figures 18 and 19 depict the impact of the disruptive chemical reaction factor ($\Upsilon$ > 0) on profiles $T_{0}$ and $T_{1}$. The concentration profile $T_{0}$ and $T_{1}$ are revealed to be a decreasing function of $\Upsilon$. It's also evident that as $\Upsilon$ improves, the strength of $T_{0}$ and $T_{1}$ reduce.

Figures 20 and 21 reveal how the concentration profiles $T_{0}$ and $T_{1}$ change as a generating chemical reaction ($\Upsilon$ < 0) progresses. On the other hand, when $T_{0}$ grows for large generating chemical reaction parameters, as seen in Fig. 18. The amplitude of $T_{1}$ grows as $\Upsilon$ ($\Upsilon$ < 0) grows, as shown in Fig. 21.

The effect of Sc and $\Upsilon$ on mass transfer $T_{0}^{\prime } (0)$ and $T_{1}^{\prime } (0)$ is shown in Figs. 22 and 23. It is revealed that the superficial mass transfer $T_{0}$ enhances while $T_{1}$ reduces as Sc and $\Upsilon$ are increasing. Figures 24 and 25 show the influence of $M$ and Sc on the $T_{0}^{\prime } (0)$ and $T_{1}^{\prime } (0).$ From these figures, it is clear form this figure that as $M$ increases, the surface mass transfer $T_{0}$ decreases and $T_{1}$ enhances.

Table 1 shows the variation of surface mass transfer $- T_{0}^{\prime } (0)$ and $- T_{1}^{\prime } (0)$ for some values of $Sc$, $\Upsilon$, $M$, and $\Omega_{1} = \Omega_{2}$. From this table, it is observed that the values mass transfer $- T_{0}^{\prime } (0)$ increases for $Sc$, $\Upsilon$, and $\Omega_{1} = \Omega_{2}$ whereas declines for increasing $M$. Similarly, the amount of $- T_{1}^{\prime } (0)$ enhances with the increasing values of $Sc$, $M$, and $\Omega_{1} = \Omega_{2}$ and reduces for large values of $\Upsilon$. For confirmation of the present work calculation, the existing work is also compared with published work reported by Liao^21^ for surface mass transfer $- T_{0}^{\prime } (0)$ and $- T_{1}^{\prime } (0)$, and excellent agreement is found as shown in table below.

## Closing remarks

The mass transfer inside the magnetohydrodynamics free convection movement of a Jeffrey liquid limited by a stretching/shrinking surface is examined in this paper. The concentration and velocity profiles are calculated. For the numerical solutions, the Runge–Kutta fourth order method is used. For the conformation of code bvp4c is also applied and excellent agreement is found The behaviour of numerous entrenched factors in the modelling under consideration is investigated. In tabular formats, the gradient of mass transfer is also estimated. The most important points are highlighted here.

1. On $f^{\prime } {\text{ and }}g^{\prime }$, the performance of $\Omega_{1}$ and $M_{1}$ is same.
2. The boundary-layer thickness is reduced when the value of $M_{1}$ is increased.
3. As Sc grows, the concentration fields $T_{0}$ and $T_{1}$ decline.
4. The destructive response ($\Upsilon$ > 0) has the effect of lowering the concentration profiles.
5. For destructive ($\Upsilon$ > 0) and generative ($\Upsilon$< 0) chemical reactions, the concentration fields $T_{0}$ and $T_{1}$ observed opposite effects.
6. By rising $M$, the surface mass transfer reduces.

## Data Availability

The database used and analysed during the current study are available from the corresponding author on reasonable request.
